# Bedside Small Bowel Follow-Through: The Role in the Management of Adhesive Small Bowel Obstruction

**DOI:** 10.7759/cureus.74027

**Published:** 2024-11-19

**Authors:** Lindsay A Duy, Pinyu Chen, Sean K Wang, Michael Y Chen, Preston R Miller, W.T. Hillman Terzian, Raymond B Dyer

**Affiliations:** 1 Department of Radiology, Atrium Health Wake Forest Baptist Medical Center, Winston-Salem, USA; 2 Department of Surgery, Atrium Health Wake Forest Baptist Medical Center, Winston-Salem, USA; 3 Department of Surgery, University of Nebraska Medical Center, Omaha, USA

**Keywords:** abdominal radiograph, adhesive small bowel obstruction, colonic contrast, gastrografin challenge, post-operative management

## Abstract

Background: Adhesive small bowel obstruction (ASBO) accounts for the majority of hospitalizations related to SBO following abdominal surgery. Delays in the management of ASBO are associated with longer hospital stays and increased mortality rates, making it imperative to establish an efficient way of determining which patients need surgical intervention.

Purpose: To evaluate the contribution of bedside small bowel follow-through (BSBFT) in the management of suspected ASBO.

Materials and methods: A single-site analysis of 320 patients who underwent BSBFT from August 2015 to 2019 was retrospectively performed. The presence of contrast in the colon on abdominal radiographs obtained at eight and 24 hours after administration and subsequent management (conservative versus surgical) was recorded.

Results: Of the 320 BSBFT exams, 235 cases had colonic contrast present at eight hours. Twelve of those cases received surgical treatment, while the remaining 223 were managed conservatively. Forty-three cases showed colonic contrast at 24 hours despite not showing contrast at eight hours. Of these cases, 29 patients were managed conservatively, while 14 patients underwent surgery. Forty-two cases had no contrast at 24 hours, and 33 patients of those patients subsequently received surgical intervention, while nine were managed conservatively. Patients who had contrast on radiographs and underwent surgical interventions had either high clinical concern for postoperative complications or stagnant clinical progression.

Conclusion: BSBFT helps determine the management for suspected ASBO. ​Patients with colonic contrast on eight- or 24-hour abdominal images were more likely to be managed conservatively. However, the clinical context is important, since clinical factors may overrule the results of the BSBFT.

## Introduction

Small bowel obstruction (SBO) is a common cause of hospital admission in the United States, accounting for 15% of acute abdominal pain cases and 20% of acute surgical care cases [1-4]. Accounting for 54-75% of SBO cases, adhesive SBO (ASBO) is the main etiology of SBO and the major cause of SBO operations [5-7]. Despite the increased use of anti-adhesion barriers over the years, there is still a steady rise in hospitalizations for ASBO [8]. Developing postoperative intra-abdominal adhesions following abdominal surgery is almost definite with 2-9% of these patients ultimately developing SBO [4,7,9]. Adhesiolysis procedures are responsible for more than 300,000 hospitalizations a year, which translates to 1% of hospitalizations nationwide; 846,000 inpatient care days; and $1.33 billion dollars annually in hospital and surgical expenditures. Recently, inpatient expenditures have decreased by nearly 10% as a result of a 15% decrease in the average hospital length of stay (LOS) [10]. SBO patients generally stay in the hospital for 4-13 days [7,11]. These expenditures, however, do not factor in indirect cost expenditures for patients, such as time off work or decreased productivity because of long-term complications [10]. Because of the staggering costs associated with ASBO, it is necessary to investigate management strategies to decrease LOS and financial burden.

Delays in the management of ASBO are associated with longer hospital LOS and increased mortality rates​[11,12]. Patients not operated on within three days of hospitalization had an increased 30-day mortality rate, and patients not operated on within four days of hospitalization had an increase in postoperative LOS [11]. In some reports, treatment delay of more than 24 hours for ASBO is associated with a 5% increased risk of death and postoperative complications [6,11]. There is a lack of consensus about how long surgeons should wait before operating on a patient with ASBO [13-16]. Originally, the thought process was that a surgeon should “never let the sun rise and set on an SBO”; however, there has been a shift to conservative, non-operative treatment when appropriate [13-15]. Development of bowel incarceration and ischemia is indicative of a need for operative management [17]. Patients managed conservatively for SBO have an average stay of four days in the hospital, compared to seven days for operative treatment [15].

Improvements in imaging techniques for ASBO have helped with ASBO evaluation and determination of treatment plan [4,14]. Bedside small bowel follow-through (BSBFT), otherwise known as the Gastrografin® challenge, allows for earlier recognition of complete obstruction, lowers rates of operative exploration, and decreases LOS [17-20]. Gastrografin® is a water-soluble, hyperosmotic oral contrast agent, making it a useful diagnostic tool in the management of ASBO [17]. Several studies have indicated therapeutic effects associated with the use of Gastrografin® in patients; however, the results are still open to debate [17,21]. There is a direct relationship between the amount of time that it takes Gastrografin® to reach the colon and the LOS [12]. Although the benefits of using Gastrografin® have been reported, there is no standard protocol for how long to wait for contrast to reach the colon before deciding on surgical management [12].

To our knowledge, this report reviews the largest single-site radiology study of the outcome (conservative or surgical management) after BSBFT. The purpose of this study is to evaluate the contribution of the BSBFT, also known as the Gastrografin® challenge in the management of patients presenting with suspected adhesive small bowel obstruction.

## Materials and methods

This Health Insurance Portability and Accountability Act (HIPAA)-compliant retrospective single-site study was approved by the Wake Forest University Health Sciences Institutional Review Board (IRB00029848). We retrospectively reviewed 1,000 patient charts and imaging studies from a four-year span through WakeOne (Epic Systems, Verona, WI) and IntelliSpace PACS (Philips, New York, NY), respectively. No patients were contacted in this study. All patients seen at an academic medical center and health system from August 2015 to August 2019, who had undergone BSBFT, were initially evaluated. All patients who had a BSBFT performed by the Department of Radiology were included in this study regardless of gender, race, age, and health status. Cases were excluded if an eight-hour abdomen image was not performed, did not show any visible enteric contrast, was performed in duplicate, or was equivocal. Additional exclusion criteria included cases in which a 24-hour abdomen image was not performed or did not show enteric contrast. If a patient met all the imaging inclusion criteria, their medical record was reviewed to determine how they were managed during their post-operative stay, as well as their clinical outcomes.

**Figure 1:**
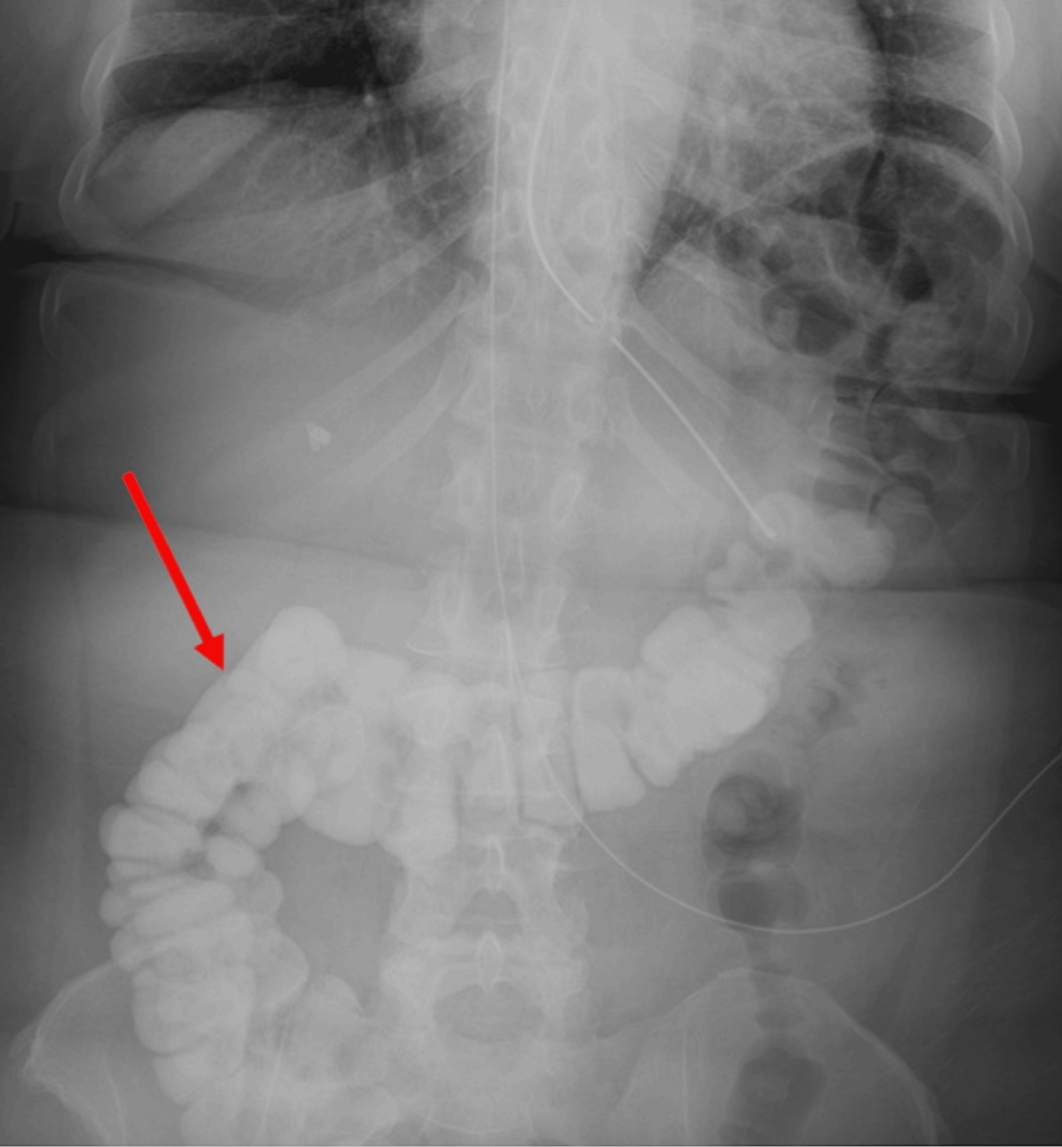
An abdominal image obtained eight hours after NG contrast administration shows contrast in the colon (red arrow).

**Figure 2:**
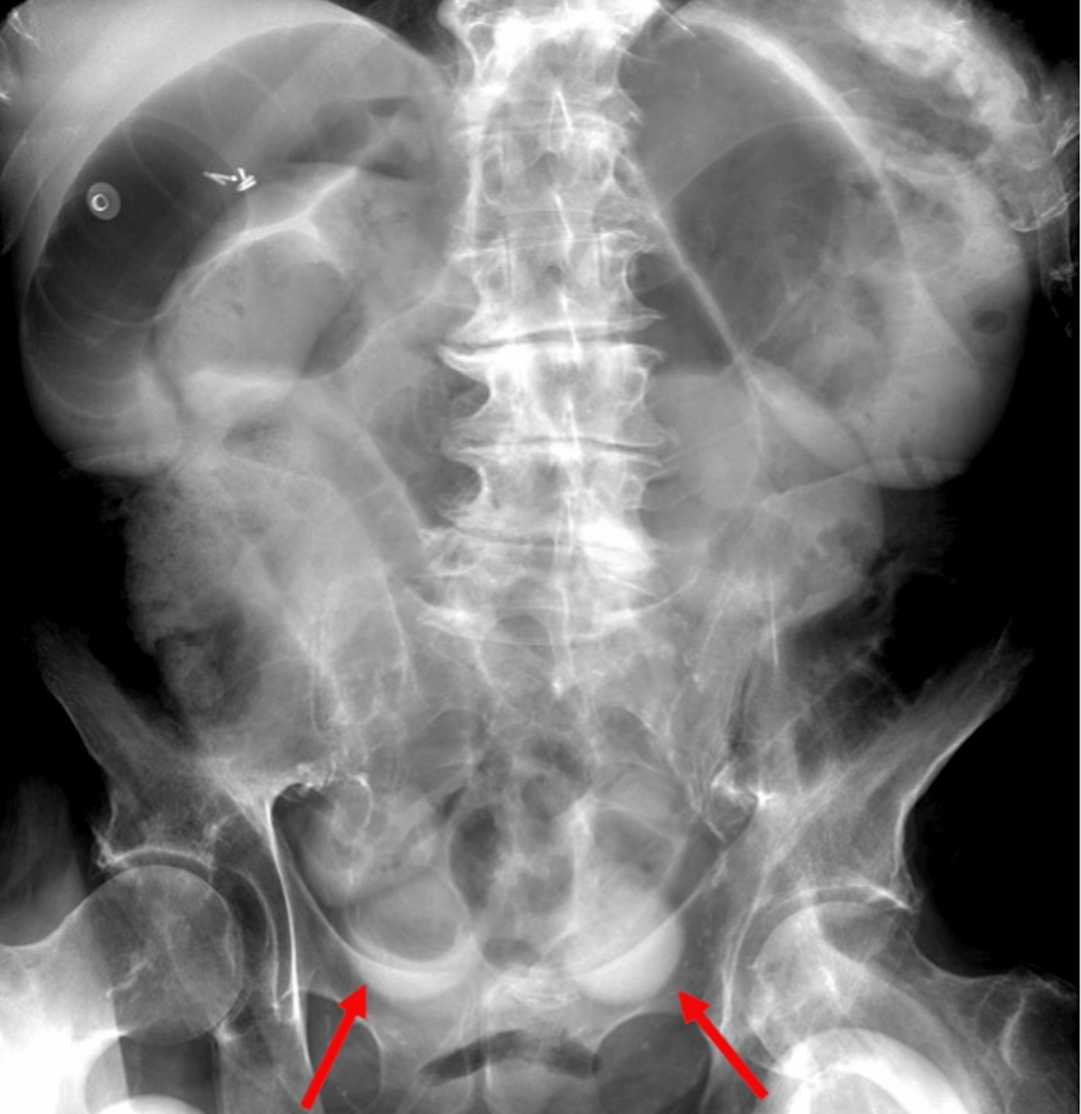
An abdominal image obtained eight hours after NG contrast administration shows contrast in dilated small bowel loops (red arrows). No colonic contrast is visualized.

The protocol that was followed for BSBFT was as follows. Gastric decompression with a nasogastric (NG) tube was performed 24 hours prior to contrast administration where possible, after which 120 mL of a hyperosmolar, water-soluble contrast agent was administered through the NG tube to each patient. The NG tube was then clamped for one hour. An abdominal radiograph was obtained eight hours after contrast administration. Patients who had colonic contrast visualized on the eight-hour radiograph (Figure 1) had no further imaging. Patients who did not show colonic contrast on the eight-hour image had an additional abdominal radiograph 24 hours after contrast administration (Figure 2).

## Results

Initially, 379 cases of BSBFT from August 2015 to August 2019 were enrolled. However, 59 cases were excluded. Thirty-four cases were excluded because the eight-hour abdominal image was not performed, did not show any enteric contrast, was performed in duplicate, or was equivocal. Fourteen cases were excluded because the 24-hour abdominal image was not performed. Eleven cases were excluded because the 24-hour abdominal image failed to show any enteric contrast.

**Figure 3:**
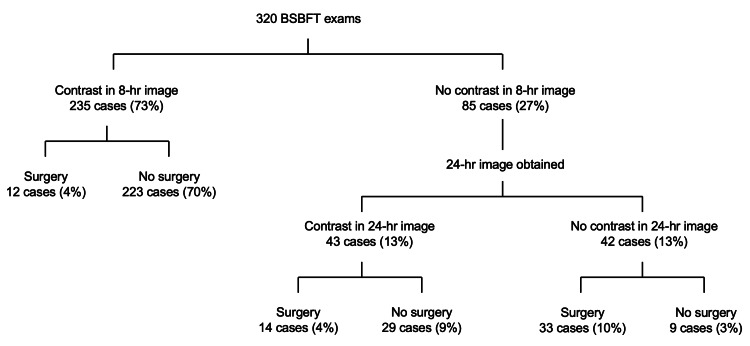
Organizational chart of patient outcomes.

In total, 320 BSBFT exams (Figure 3) from 299 patients with an average age of 63 years (range of 18-96 years) were reviewed for this study (Table 1). Of these patients, 137 (46%) were male. In 14 cases, the same patient was admitted twice. In two cases, the same patient was admitted three times. One patient was admitted four times.

**Table 1:** Patient demographic data.

Total Number of Patients	Average Age (yrs)	Age Range (yrs)	Male	Female
299	18-96	63	137 (46%)	162 (54%)

Of the cases reviewed, 235 (73%) patients had colonic contrast on the eight-hour abdominal image (Figure 1). Patients in 12 of these cases (4%) underwent operative intervention, while patients in 223 of these cases (70%) were managed conservatively.

For the 85 cases (27%) without colonic contrast on the eight-hour abdominal image (27%), an abdominal radiograph was taken 24 hours after contrast administration. Forty-three of these cases had contrast on 24-hour abdominal radiographs (13%). Of these patients, 14 cases (4%) underwent surgery, while patients in the remaining 29 cases were managed conservatively (9%). In the 42 cases (13%) where colonic contrast was not noted on the 24-hour abdominal image (13%), patients in 33 of these cases (10%) underwent surgery, while patients in the remaining nine cases (3%) were managed conservatively.

In the 12 cases where the patients underwent surgery despite visible colonic contrast on the eight-hour radiograph, surgery was performed because the obstruction appeared non-adhesive on CT or there was clinical concern for associated complications. In the 14 cases where the patients underwent surgery despite visible colonic contrast on the 24-hour radiograph, surgery was performed because most of the patients had no clinical progression. In the nine cases managed conservatively, despite no visualized colonic contrast on the 24-hour abdominal image, there was a clinical improvement or a high clinical suspicion of ileus.

## Discussion

BSBFT can play an important role in assisting with the management decisions for suspected ASBO. ​Based on this study cohort, patients who show colonic contrast on eight-hour or 24-hour abdominal images following hyperosmolar enteric contrast administration can more likely be managed conservatively. ​This test is less useful in patients who have a non-adhesive etiology for obstruction or in patients who are poor surgical candidates. ​Close clinical monitoring and ancillary information provided by CT remain crucial to management decisions if there is clinical concern for complications, such as a closed-loop obstruction or bowel ischemia. Surgery may still be indicated in some patients who show colonic contrast within this timeframe if these patients fail to clinically improve.​ Currently, this is the largest single-site radiology study examining the impact of BSBFT on the management of patients with suspected ASBO.

Previous studies have produced similar BSBFT findings (Table 2). Loftus et al. investigated the use of BSBFT to differentiate between partial ASBO or complete obstruction; patients were given contrast and abdominal plain radiographs were taken at four, eight, 12, and 24 hours after contrast administration. Of the 72 patients in the BSBFT group, 43% of the patients had no colon contrast at 24 hours and were all taken to surgery [12]. Compared to our study, only 13% of the patients did not have contrast at 24 hours, and out of this group, 79% went to surgery. This difference may be accounted for by clinical factors. In our study, patients who did not undergo surgery showed clinical improvement or there was a high suspicion for ileus. Similarly, there were patients who had colon contrast on 24-hour images after contrast administration but still ended up going to surgery [12]. As indicated by the Bologna Guidelines for the management of ASBO, even if patients had contrast in the colon, they should be managed surgically if there are other indications, such as clinical failure of conservative management or concern for complications such as bowel ischemia or peritonitis [22]. Another study looking at the Gastrografin® challenge by Goussous et al., where colonic contrast was present on eight-hour images after administration, had more comparable findings to this study’s results. Of the patients who went through the Gastrografin® challenge, 83% of 53 patients had contrast in the colon after eight hours. Of the subset of patients who had contrast, 11% of them still underwent surgery, compared to the 4% of total patients in this current study [19].

**Table 2:** Comparison of the percentage of patients who had surgery at the specified timepoint with and without contrast in the colon.

Research Article	Year	With Contrast in Colon	Without Contrast in Colon	Timepoint of Surgery Intervention
Duy et al.	2024	26/278 (9%)	33/42 (79%)	8 hrs, 24 hrs
Mulder et al. [23]	2019	0/208 (0%)	78/78 (100%)	96 hrs
Scotté et al. [24]	2017	0/92 (0%)	29/29 (100%)	48 hrs
Zielinski et al. [17]	2017	9/118 (7.6%)	31/55 (59%)	8 hrs
Loftus et al. [12]	2015	0/41 (0%)	31/31 (100%)	24 hrs
Weiss et al. [25]	2017	4/124 (3%)	20/30 (67%)	24 hrs
Kuehn et al. [26]	2017	0/85 (0%)	6/20 (30%)	12 hrs, 24 hrs
Mori et al. [27]	2017	12/659 (2%)	117/117 (100%)	5 hrs
Goussous et al. [19]	2013	5/44 (11%)	8/9 (89%)	8 hrs
Atahan et al. [28]	2010	0/24 (0%)	10/13 (77%)	8 hrs
Farid et al. [29]	2010	1/48 (2%)	7/7 (100%)	48 hrs

Our study had two major limitations. First, as a retrospective study, we were only able to observe association, rather than causality. Furthermore, there is an absence of longitudinal follow-up looking at the patient outcomes, such as an increased one-year ASBO recurrence rate in patients without visible enteric contrast on the 24-hour radiograph [30]. This issue could be further evaluated by conducting a prospective study with at least one year of follow-up. Second, because patients were not matched to control, it is unclear what the patient outcome would have been had they not received surgical intervention. It is possible their imaging would have shown colonic contrast at a later timepoint. Mulder et al. extended non-operative management to patients showing colonic contrast beyond 48 hours during BSBFT to reduce the need for surgery [23].

## Conclusions

Across various studies and institutions, there is no standard BSBFT protocol, particularly with imaging timepoints. Looking ahead, a future direction for investigation is the development of a protocol with decompression time and imaging timepoints that would be diagnostic, safe, and cost-effective and potentially reduce hospital LOS. BSBFT is a useful tool and can assist the surgeon with management decisions in ASBO. Clinical context remains a major factor when determining treatment for these patients. Radiology departments should have a BSBFT protocol in place to facilitate the care of patients with ASBO.
